# Existence of anti-periodic (differentiable) mild solutions to semilinear differential equations with nondense domain

**DOI:** 10.1186/s40064-016-2315-1

**Published:** 2016-06-10

**Authors:** Jinghuai Liu, Litao Zhang

**Affiliations:** Department of Mathematics and Physics, Zhengzhou University of Aeronautics, Zhengzhou, 450015 People’s Republic of China; Collaborative Innovation Center for Aviation Economy Development of Henan Province, Zhengzhou University of Aeronautics, Zhengzhou, 450015 People’s Republic of China

**Keywords:** Anti-periodic (differentiable) function, Mild solutions, Hille–Yosida operators, Semilinear differential equations, 35B10, 47D06

## Abstract

In this paper, we investigate the existence of anti-periodic (or anti-periodic differentiable) mild solutions to the semilinear differential equation $u'(t) = Au(t) + f (t, u(t))$ with nondense domain. Furthermore, an example is given to illustrate our results.

## Background

In this paper, we study the existence of anti-periodic (or anti-periodic differentiable) mild solutions to the semilinear differential equation

1 $$\begin{aligned} u'(t) = Au(t) + f (t, u(t)),\quad t\in R \end{aligned}$$

where *A* is an unbounded linear operator, assumed to be Hille–Yosida of negative $\omega$-type having the domain D(A) not necessarily dense in some Banach spaces *X* (for more details, see “Preliminaries” section), and $f:R\times X_{0}\rightarrow X$ is a suitable function.

The problem about the existence of anti-periodic solutions constitutes one of the most attractive topics in qualitative theory of differential equations due to its applications in engineering, physics, control theory and other subjects. We refer to the works of Aftabizadeh et al. (1994), Al-Islam et al. (2012), Aizicovici et al. (2001), Cao et al. (2012), Chen et al. (2007), Haraux (1989), Liu et al. (2015), Liu et al. (2015), N’Guérékata and Valmorin (2012), Okochi (1990), Wang and Chen (2013), Yang and Srivastava (2015), Abdurahman and Jiang (2015), Xu (2016), Chadli et al. (2016) and the references therein. However, most of these problems need to be studied in abstract spaces and operators are defined over nondense domain. The literature concerning the existence of anti-periodic solutions for differential equations with nondense domain is new.

To the best of our knowledge, the existence of anti-periodic (or anti-periodic differentiable) mild solutions for semilinear differential equation with nondense domain constitutes until now an untreated original problem. This fact is the main motivations of this paper. To illustrate our abstract results, the existence and uniqueness of anti-periodic solutions to a partial differential equation is discussed.

The paper is organized as follows: In “Preliminaries” section, we give some definitions and fix notations which will be used in the sequel. In “Main results and proofs” section, the existence of anti-periodic (or anti-periodic differentiable) mild solution to some semilinear differential equations in Banach space are studied. In “An example” section, an example is given to illustrate our main results.

## Preliminaries

In this section we recall some definitions and fix notations which will be used in the sequel. We assume that *X* is a Banach space endowed with the norm $\Vert \cdot \Vert$ and *B*(*X*) stands for the Banach space of all bounded linear operators from *X* to itself. $R^{+}$ denotes the set of nonnegative real numbers. $C_{b}(R,X)$ denotes the space of all bounded continuous functions from $R\rightarrow X$. Moreover, we denote by $C^{1}(R,X)$ the space of all functions $R\rightarrow X$ which have a continuous derivative on *R*. $C_{b}^{1}(R,X)$ is the subspace of $C^{1}(R,X)$ consists of such functions satisfying

$$\begin{aligned} \sup _{t\in R}(\Vert f(t)\Vert +\Vert f'(t)\Vert )<\infty . \end{aligned}$$

It is clear that $C_{b}^{1}(R,X)$ turns out to be a Banach space with the norm

$$\begin{aligned} \Vert f\Vert _{C_{b}^{1}(R,X)}=\sup _{t\in R}(\Vert f(t)\Vert +\Vert f'(t)\Vert ). \end{aligned}$$

We first recall some properties of Hille–Yosida operators and extrapolation spaces. For more details, we refer to Amir and Maniar (1999), Agarwal et al. (2011), Prato and Grisvard (1982), Engel and Nagel (2001), Hille and Philips (1975), Nagel and Sinestrari (1994) and the references therein.

### **Definition 1**

(Agarwal et al. 2011) Let *A* be a linear operator with domain *D*(*A*). We say that (*A*, *D*(*A*)) is a Hille–Yosida operator on *X* if there exist $\omega \in R$ and a positive constant $M\ge 1$ such that $(\omega , \infty )\subseteq \rho (A)$ and $\sup \{(\lambda -\omega )^{n}\Vert (\lambda -A)\Vert ^{-n}\}\le M$. The infinimum of such a $\omega$ is called the type of *A*. If the constant $\omega$ can be chosen smaller than zero, *A* is said to be of negative type.

From the Hille–Yosida theorem (Engel and Nagel 2001, Theorem II.3.8) we have the following result.

### **Lemma 1**

*Let* (*A*, *D*(*A*)) *be a Hille–Yosida operator onX*, $X_{0}=\overline{D(A)}$, $D(A_{0})=\{x\in D(A):Ax\in X_{0}\}$*and*$A_{0}:D(A_{0})\subset X_{0}\rightarrow X_{0}$*be the operatordefined by*$A_{0}x=Ax.$*The operator*$A_{0}$*generates a*$C_{0}$*semigroup*$(T_{0}(t))_{t\ge 0}$*on*$X_{0}$*with*$\Vert T_{0}(t)\Vert \le Me^{\omega t}$*for*$t\ge 0$. *Moreover,*$\rho (A)\subset \rho (A_{0})$*and*$R(\lambda ,A_{0})=R(\lambda ,A)|_{X_{0}}$, *for*$\lambda \in \rho (A)$.

Let $\lambda \in \rho (A)$. we define a norm on space $X_{0}$ by

$$\begin{aligned} \Vert x\Vert _{-1}=\Vert R(\lambda ,A_{0}) x\Vert , \quad x \in X_{0}. \end{aligned}$$

The completion of $(X_{0},\Vert \cdot \Vert _{-1})$ will be called the extrapolation space of $X_{0}$ associated with $A_{0}$ and will be denoted by $X_{-1}$. One can show easily that, $T_{0}(t)$ has a unique bounded linear extension $T_{-1}(t)$ to $X_{-1}$. The operator family $(T_{-1}(t))_{t\ge 0}$ is a $C_{0}$ semigroup on $X_{-1}$, called the extrapolated semigroup of $(T_{0}(t))_{t\ge 0}$. The domain of its generator $A_{-1}$ is equal to $A_{0}$.

The following results have been established in Amir and Maniar (1999), Agarwal et al. (2011), Nagel and Sinestrari (1994).

### **Lemma 2**

*Under the previous conditions, the following properties are verified.*

(i) $D(A_{-1})=X_{0}$*and*$\Vert T_{-1}(t)\Vert _{B(X_{-1})}=\Vert T_{0}(t)\Vert _{B(X_{0})}$*for every*$t\ge 0$;
(ii) *The operator*$A_{-1}:X_{0}\rightarrow X_{-1}$*is the uniquecontinuous extension of*$A_{0}:D(A_{0})\subset (X_{0},\Vert \cdot \Vert )\rightarrow (X_{-1},\Vert \cdot \Vert _{-1})$*and*$\lambda -A_{-1}$*is an isometry from*$(X_{0},\Vert \cdot \Vert )$*to*$(X_{-1},\Vert \cdot \Vert _{-1})$;
(iii) *If*$\lambda \in \rho (A_{0})$, *then*$(\lambda -A_{-1})^{-1}$*exists and*$(\lambda -A_{-1})^{-1}\in B(X_{-1})$. *In particular,*$\lambda \in \rho (A_{-1})$*and*$R(\lambda ,A_{-1})|_{X_{0}}=R(\lambda ,A_{0})$;
(iv) *The space*$X_{0}=\overline{D(A)}$*is dense in*$(X_{-1},\Vert \cdot \Vert _{-1})$. *Thus, the extrapolation space*$X_{-1}$*isalso the completion of*$(X,\Vert \cdot \Vert _{-1})$*and*$X\hookrightarrow X_{-1}$. *Moreover,*$A_{-1}$*is an extension ofA* to $X_{-1}$. *In particular, if*$\lambda \in \rho (A)$, *then*$R(\lambda ,A_{-1})|_{X}=R(\lambda ,A)$*and*$R(\lambda ,A_{-1})=D(A)$.

### **Lemma 3**

$f\in C_{b}(R,X)$*and* (*A*, *D*(*A*)) *be a Hille–Yosida operator of negative*$\omega$*-type. Then the following properties are valid.*

(i) $$\int _{-\infty }^{t}T_{-1}(t-s)f(s)ds\in X_{0}$$, *for every*$$t\in R$$;
(ii) $\Vert \int _{-\infty }^{t}T_{-1}(t-s)f(s)ds\Vert \le Me^{\omega t}\int _{-\infty }^{t}e^{-\omega s}\Vert f(s)\Vert ds$*where*$M>0$*is independent oftandf*;
(iii) *The function*$\Gamma (f)(t)=\int _{-\infty }^{t}T_{-1}(t-s)f(s)ds\in X_{0}$*iscontinuous, where the operator*$\Gamma :C_{b}(R,X)\rightarrow C_{b}(R,X_{0})$.

Now, we recall a useful compactness criterion.

Let $h:R\rightarrow R$ be a continuous function such that $h(t)\ge 1$ for all $t\in R$, and $h(t)\rightarrow \infty$ as $|t|\rightarrow \infty$. We consider the space

$$\begin{aligned} C_{h}(X)=\left\{u\in C(R,X):\lim _{|t|\rightarrow \infty }\frac{u(t)}{h(t)}=0\right\} \end{aligned}$$

endowed with the norm

$$\begin{aligned} \Vert u\Vert _{h}=\sup _{t\in R}\frac{\Vert u(t)\Vert }{h(t)}. \end{aligned}$$

### **Lemma 4**

(Henríquez and Lizama 2009) *A subset*$K \subseteq C_{h}(X)$*is a relatively compact set if it verifies the following conditions:*

(i) *The set*$K(t)=\{u(t):u\in K\}$*is relatively compact inXfor each*$t\in R$;
(ii) *The setKis equicontinuous;*
(iii) *For each*$\varepsilon >0$*there exists*$L>0$*such that*$\Vert u(t)\Vert \le \varepsilon h(t)$*for all*$u\in K$*and all*$|t|>L$.

### *Remark 1*

(Henríquez and Lizama 2009) It is clear that $C_{h}(X)$ is a Banach space isometrically isomorphic with the space $C_{0}(R,X)$ consisting of functions that vanish at infinity.

Also, we recall some notations about Stepanov bounded functions and anti-periodic functions.

### **Definition 2**

(Pankov 1990) The Bochner transform $f^{b}(t,s)$, $t\in R$, $s\in [0,1]$, of a function *f*(*t*) on *R*, with values in *X*, is defined by

$$\begin{aligned} f^{b}(t,s):=f(t+s). \end{aligned}$$

### **Definition 3**

(Pankov 1990) Let $p\in [1,\infty )$. The space $BS^{p}(X)$ of all Stepanov bounded functions, with the exponent *p*, consists of all measurable functions *f* on *R* with values in *X* such that $f^{b}\in L^{\infty }(R,L^{p}(0,1;X))$. This is a Banach space with the norm

$$\begin{aligned} \Vert f\Vert _{S^{p}}=\Vert f^{b}\Vert _{L^{\infty }(R,L^{p})}=\sup _{t\in R}\left( \int _{t}^{t+1}\Vert f(\tau )\Vert ^{p}d\tau \right) ^{1/p}. \end{aligned}$$

### **Definition 4**

(Aizicovici et al. 2001) A function $f\in C_{b}(R,X)$ is called anti-periodic provided that

$$\begin{aligned} f(t+T)=-f(t), \quad \forall t\in R. \end{aligned}$$

Denote by $P_{TA}(R,X)$ the set of all anti-periodic functions.

### **Lemma 5**

(N’Guérékata and Valmorin 2012) *Let*$f_{n}\in P_{TA}(R,X)$, *such that*$f_{n}\rightarrow f$*uniformly on R. Then*$f\in P_{TA}(R,X)$.

### **Lemma 6**

(N’Guérékata and Valmorin 2012) *The*$P_{TA}(R,X)$*is a Banach space equipped with the supnorm.*

### **Lemma 7**

(N’Guérékata and Valmorin 2012) *If*$f\in C^{1}(R,X)$*is anti-periodic, then*$f'\in P_{TA}(R,X)$.

### **Definition 5**

A continuous function *f* is said to be anti-periodic differentiable if $f\in P_{TA}(R,X)$ and $f'\in P_{TA}(R,X)$.

Denote by $P'_{TA}(R,X)$ the set of all such functions.

### **Lemma 8**

(Liu et al. 2015) $P'_{TA}(R,X)$*is a Banach space with the supremum norm given by*

$$\begin{aligned} \Vert f\Vert _{P'_{TA}(R,X)}= \sup _{t\in R}(\Vert f(t)\Vert +\Vert f'(t)\Vert ). \end{aligned}$$

### **Definition 6**

(Amir and Maniar 2000) *Let* (*A*, *D*(*A*)) be a Hille–Yosida operator of negative $\omega$-type. A function $u(t): R\rightarrow X$ satisfying the equation

$$\begin{aligned} u(t)=T_{0}(t-s)u(s)+ \int _{s}^{t}T_{-1}(t-\tau )f(\tau ,u(\tau ))d\tau , \end{aligned}$$

for all $t\ge s>-\infty$ is called a mild solution of semilinear differential equation

$$\begin{aligned} u'(t) = Au(t) + f (t, u(t)), t\in R. \end{aligned}$$

We give the famous Schauder’s fixed point theorem as follows:

### **Lemma 9**

(Smart 1980) *LetDbe a nonempty, closed, bounded, convex subset of a Banach spaceX*. *Let*$F:D\rightarrow D$*be a continuous and compact operator, then the operator equation*$Fu=u$*has a fixed point inD*.

## Main results and proofs

In this section, we study the existence of anti-periodic (or anti-periodic differentiable) mild solutions of Eq. (1). The following are the main results of this work.

First, we list some assumptions.

$$(H_{1})$$
The function $f:R\times X_{0}\rightarrow X$ is continuous and $f(t+T,-u)=-f(t,u)$ for all $t\in R$, $u\in X_{0}$
$$(H_{2})$$
The function $f:R\times X_{0}\rightarrow X$ satisfies the condition:
  $$\begin{aligned} \Vert f(t,x)-f(t,y)\Vert \le L(t)\Vert x-y\Vert \end{aligned}$$
  for all $t\in R$, $x,y\in X_{0}$, where $L(t)\in BS^{p}(R)$.

The following theorem is needed to establish our next results.

### **Theorem 1**

*Let* (*A*, *D*(*A*)) *be a Hille–Yosida operator of negative*$\omega$*-type andfsatisfy the condition*$(H_{1})$. *The*$\Gamma :C_{b}(R,X)\rightarrow C_{b}(R,X_{0})$*is a linear operator and*$\Gamma u(t)$*is defined by*

$$\begin{aligned} \Gamma u(t)=\int _{-\infty }^{t}T_{-1}(t-s)f(s,u(s))ds \end{aligned}$$

*for every*$$t\in R$$.

*If*$$u\in P_{TA}(R,X_{0})$$*, then*$$\Gamma u(t)\in P_{TA}(R,X_{0})$$.

### *Proof*

Firstly, it is easily to see that

$$\begin{aligned} \Vert \Gamma u(t)\Vert\le & {} \int _{-\infty }^{t}\Vert T_{-1}(t-s)f(s,u(s))\Vert ds \nonumber \\\le & {} \int _{-\infty }^{t}Me^{\omega (t-s)}\Vert f(s,u(s))\Vert ds\nonumber \\\le & {} \frac{M}{-\omega }\Vert f\Vert .\nonumber \end{aligned}$$

Thus $\Gamma$ is well defined and $\Gamma u$ is bounded.

Secondly, for any $t\in R,\quad h\in R$ is small enough

$$\begin{aligned} \left\Vert \Gamma u(t+h)-\Gamma u(t)\right\Vert&= {} \left\Vert \int _{-\infty }^{t+h}T_{-1}(t+h-s)f(s,u(s))ds-\int _{-\infty }^{t}T_{-1}(t-s)f(s,u(s))ds\right\Vert \nonumber \\ &= \left\Vert \int _{-\infty }^{t}T_{-1}(t-s)[f(s+h,u(s+h))-f(s,u(s))]ds\right\Vert \nonumber \\ & \le Me^{\omega t}\int _{-\infty }^{t}e^{-\omega s}\left\Vert f(s+h,u(s+h))-f(s,u(s))\right\Vert ds\nonumber \\ &\le \frac{M}{-\omega }\left\Vert f(s+h,u(s+h))-f(s,u(s))\right\Vert .\nonumber \end{aligned}$$

Thus, $\Vert \Gamma u(t+h)-\Gamma u(t)\Vert \rightarrow 0$ as $h\rightarrow 0$, which proves that $\Gamma u$ is continuous.

Finally, It follows from $(H_{1})$ that for any $u\in P_{TA}(R,X_{0})$ and for each $t\in R$

$$\begin{aligned} \Gamma u(t+T) & = \int _{-\infty }^{t+T}T_{-1}(t+T-s)f(s,u(s))ds \nonumber \\ & = \int _{-\infty }^{t}T_{-1}(t-s)f(s+T,u(s+T))ds\nonumber \\ & = -\int _{-\infty }^{t}T_{-1}(t-s)f(s,u(s))ds\nonumber \\ & = -\Gamma u(t).\nonumber \end{aligned}$$

Therefore, $\Gamma u$ is anti-periodic. The proof is complete. $\square$

### **Theorem 2**

*Let* (*A*, *D*(*A*)) *be a Hille–Yosida operator of negative*$\omega$*-type andfsatisfy the conditions*$(H_{1})$*and*$(H_{2})$. *Then Eq.* (1) *has a unique anti-periodic mild solution provided that*

$$\begin{aligned} \left( \frac{e^{\omega q}-1}{\omega q}\right) ^{\frac{1}{q}}\frac{M}{1-e^{\omega }}\Vert L\Vert _{S^{P}}<1, \quad \frac{1}{p}+\frac{1}{q}=1. \end{aligned}$$

### *Proof*

Define a operator $\Gamma$ as in Theorem 1 on $P_{TA}(R,X_{0})$ by

$$\begin{aligned} \Gamma u(t)=\int _{-\infty }^{t}T_{-1}(t-s)f(s,u(s))ds \end{aligned}$$

for every $t\in R$. By Theorem 1, the operator $\Gamma$ is well defined and maps $P_{TA}(R,X_{0})$ into itself.

Next, we prove that the operator $\Gamma$ has a unique fixed point in $P_{TA}(R,X_{0})$.

Let $$u,v\in P_{TA}(R,X_{0})$$, then

$$\begin{aligned} \left\Vert \Gamma u(t)-\Gamma v(t)\right\Vert&= \left\Vert \int _{-\infty }^{t}T_{-1}(t-s)[f(s,u(s))-f(s,v(s))]ds\right\Vert \nonumber \\ & \le M\int _{-\infty }^{t}e^{\omega (t-s)}\left\Vert f(s,u(s))-f(s,v(s))\right\Vert ds\nonumber \\ & \le M\int _{0}^{\infty }e^{\omega s}L(t-s)\left\Vert u-v\right\Vert ds\nonumber \\ & \le M\left\Vert u-v\right\Vert \sum _{k\ge 0}\int _{k}^{k+1 }e^{\omega s}L(t-s)ds\nonumber \\ & \le M\left\Vert u-v\right\Vert \sum _{k\ge 0}\left( \int _{k}^{k+1 }e^{\omega qs}ds\right) ^{\frac{1}{q}} \left( \int _{k}^{k+1 }L^{p}(t-s)ds\right) ^{\frac{1}{p}}\nonumber \\ & \le M\Vert L\Vert _{S^{P}}\Vert u-v \Vert \sum _{k\ge 0} \left( \int _{k}^{k+1 }e^{\omega qs}ds\right) ^{\frac{1}{q}}\nonumber \\ & \le \left( \frac{e^{\omega q}-1}{\omega q}\right) ^{\frac{1}{q}}\frac{M}{1-e^{\omega }}\Vert L\Vert _{S^{P}}\Vert u-v\Vert .\nonumber \end{aligned}$$

For $(\frac{e^{\omega q}-1}{\omega q})^{\frac{1}{q}}\frac{M}{1-e^{\omega }}\Vert L\Vert _{S^{P}}<1$, it follows from the Banach contraction mapping principle that $\Gamma$ admits a unique fixed point $u\in P_{TA}(R,X_{0})$.

Moreover, one can see easily that *u*(*t*) satisfies the variation of constants formula

$$\begin{aligned} u(t)=T_{0}(t-s)u(s)+ \int _{s}^{t}T_{-1}(t-\tau )f(\tau ,u(\tau ))d\tau , \end{aligned}$$

that is *u*(*t*) is a mild solution of Eq. (1). The proof is complete. $\square$

### **Theorem 3**

*Let* (*A*, *D*(*A*)) *be a Hille–Yosida operator of negative*$\omega$*-type andfsatisfy the conditions*$(H_{1})$*and*$(H_{2})$. *If*$L(t)\in BS^{1}(R)$, *then Eq.* (1) *has a unique anti-periodic mild solution provided that*$0<\frac{M}{1-e^{\omega }}\Vert L\Vert _{S^{1}}<1$.

### *Proof*

Define a operator $\Gamma$ as in Theorem 1 on $P_{TA}(R,X_{0})$ by

$$\begin{aligned} \Gamma u(t)=\int _{-\infty }^{t}T_{-1}(t-s)f(s,u(s))ds \end{aligned}$$

for every $t\in R$. By Theorem 1, the operator $\Gamma$ is well defined and maps $P_{TA}(R,X_{0})$ into itself.

Next, we prove that the operator $\Gamma$ has a unique fixed point in $P_{TA}(R,X_{0})$.

Let $$u,v\in P_{TA}(R,X_{0}),$$ then

$$\begin{aligned} \Vert \Gamma u(t)-\Gamma v(t)\Vert &= \left\Vert \int _{-\infty }^{t}T_{-1}(t-s)[f(s,u(s))-f(s,v(s))]ds\right\Vert \nonumber \\ &\le {} M\int _{-\infty }^{t}e^{\omega (t- s)}\Vert f(s,u(s))-f(s,v(s))\Vert ds\nonumber \\ &\le M\int _{-\infty }^{t}e^{\omega (t- s)}L(s)\Vert u-v\Vert ds\nonumber \\ &\le M\Vert u-v\Vert \sum _{k\ge 0}e^{\omega k}\int _{t-k-1}^{t-k}L(s) ds\nonumber \\ &\le M\Vert u-v\Vert \sum _{k\ge 0}e^{\omega k}\Vert L\Vert _{S^{1}}\nonumber \\ &\le \frac{M}{1-e^{\omega }}\Vert L\Vert _{S^{1}}\Vert u-v\Vert .\nonumber \end{aligned}$$

For $0<\frac{M}{1-e^{\omega }}\Vert L\Vert _{S^{1}}<1$, it follows from the Banach contraction mapping principle that $\Gamma$ admits a unique fixed point $u\in P_{TA}(R,X_{0})$. The proof is complete. $\square$

Let $L(\cdot )\equiv L$, then the following result is now immediate.

### **Theorem 4**

*Let* (*A*, *D*(*A*)) *be a Hille–Yosida operator of negative*$\omega$*-type. The functionfsatisfies the condition*$(H_{1})$*and the Lipschitz condition*

$$\begin{aligned} \Vert f(t,x)-f(t,y)\Vert \le L\Vert x-y\Vert \end{aligned}$$

*or all*$t\in R$, $x,y\in X_{0}$, *where*$L>0$*is a constant. If*$\frac{ML}{-\omega }<1$*and*$\omega <0$, *then the Eq.* (1) *has a unique anti-periodic mild solution.*

### *Proof*

Similar as the proof of Theorem 3, the proof is omitted. $\square$

### **Theorem 5**

*Let* (*A*, *D*(*A*)) *be a Hille–Yosida operator of negative*$\omega$*-type.The function*$f\in C^{1}_{b}(R,X_{0})$*satisfies the condition*$(H_{1})$*and*

$$\begin{aligned} \Vert f(t,x)-f(t,y)\Vert _{C^{1}_{b}(R,X_{0})}\le L(t)\Vert x-y\Vert _{C^{1}_{b}(R,X_{0})} \end{aligned}$$

*for all*$t\in R$, $x,y\in C^{1}_{b}(R,X_{0})$, *where*$L(t)\in BS^{P}(R)$. *If*

$$\begin{aligned} \left( \frac{e^{\omega q}-1}{\omega q}\right) ^{\frac{1}{q}}\frac{M}{1-e^{\omega }}\Vert L\Vert _{S^{P}}<1,\quad \frac{1}{p}+\frac{1}{q}=1, \end{aligned}$$

*then the Eq.* (1) *has a unique anti-periodic differentiable mild solution.*

### *Proof*

Consider the nonlinear operator $V: P'_{TA}(R,X_{0})\rightarrow C_{b}(R,X_{0})$ given by

$$\begin{aligned} (Vu)(t)=\int _{-\infty }^{t}T_{-1}(t-s)f(s,u(s))ds,\quad t\in R . \end{aligned}$$

Firstly, similar as the proof of Theorem 1, $V\in C_{b}(R,X_{0})$ is well defined.

Next, we will prove that $Vu, (Vu)'\in P_{TA}(R,X_{0})$. Let $g(\cdot )=f(\cdot ,u(\cdot ))$, Since $f\in C^{1}(R,X_{0})$ and satisfies condition $(H_{1})$, by Lemma 7, we have $g(t+T)=-g(t)$ and $g'(t+T)=-g'(t)$ for all $t\in R$. So

$$\begin{aligned} (Vu)(t+T)&= \int _{-\infty }^{t+T}T_{-1}(t+T-s)g(s)ds \nonumber \\ &= \int _{-\infty }^{t}T_{-1}(t-s)g(s+T)ds \nonumber \\ &= - \int _{-\infty }^{t}T_{-1}(t-s)g(s)ds \nonumber \\= & {} -(Vu)(t),\nonumber \end{aligned}$$

we have $Vu\in P_{TA}(R,X_{0})$. Furthermore,

$$\begin{aligned} (Vu)'(t)=\int _{-\infty }^{t}T_{-1}(t-s)g'(s)ds,\ t\in R , \end{aligned}$$

then $$(Vu)'\in P_{TA}(R,X_{0})$$.

Finally, we take $u,v\in P'_{TA}(R,X_{0})$, then we have

$$\begin{aligned} \Vert (Vu)(t)-(Vv)(t)\Vert _{P'_{TA}(R,X_{0})}&= \left\Vert \int _{-\infty }^{t}T_{-1}(t-s)[f(s,u(s))-f(s,v(s))]ds\right\Vert _{P'_{TA}(R,X_{0})}\nonumber \\ &\le \int _{-\infty }^{t}\Vert T_{-1}(t-s)\Vert L(s)\Vert u(s)-v(s)\Vert _{P'_{TA}(R,X_{0})}ds \nonumber \\ &\le M\Vert u-v\Vert _{P'_{TA}(R,X_{0})}\sum _{k\ge 0}\int _{k}^{k+1 }e^{\omega s}L(t-s)ds\nonumber \\ &\le {} \left( \frac{e^{\omega q}-1}{\omega q}\right) ^{\frac{1}{q}}\frac{M}{1-e^{\omega }}\Vert L\Vert _{S^{P}}\Vert u-v\Vert _{P'_{TA}(R,X_{0})},\nonumber \end{aligned}$$

which prove that *V* is a contraction. Hence, by using Banach contraction mapping principle that *V* admits a unique fixed point $u\in P'_{TA}(R,X_{0})$. The proof is complete. $\square$

We next study the existence of anti-periodic mild solutions of Eq. (1) when the function f is not Lipschitz continuous. To abridge the text, We assume that $f:R\times X_{0}\rightarrow X$ satisfies the following conditions:

$(A_{1})$ There is a continuous nondecreasing function $W:R^{+}\rightarrow R^{+}$, such that

$$\begin{aligned} \Vert f(t,x)\Vert \le W(\Vert x\Vert ) \end{aligned}$$

for all $$t\in R$$, $$x\in X_{0}$$;

$(A_{2})$ For each $\kappa \ge 0$, let $\beta (\kappa )=\int _{-\infty }^{t}e^{\omega (t-s)}W(\kappa h(s))ds\in C_{b}(R)$ and $M\beta (\kappa )\le r$;

$(A_{3})$ For each $\epsilon >0$, there is a $\delta >0$, such that for every $u,v\in C_{h}(X_{0})$, $\Vert u-v\Vert _{h}\le \delta$ implies

$$\begin{aligned} \sup _{t\in R}\int _{-\infty }^{t}e^{\omega (t-s)}\Vert f(s,u)-f(s,v)\Vert ds\le \epsilon ; \end{aligned}$$

$$(A_{4})$$$$T_{0}(t)$$ is a strongly continuous $$C_{0}$$ semigroup. Moreover, $$T_{0}(t)$$ is compact.

### **Theorem 6**

*Let* (*A*, *D*(*A*)) *be a Hille–Yosida operator of negative*$\omega$*-type.The functionfsatisfies the conditions*$(H_{1})$, $(A_{1})-(A_{4})$, *then Eq.* (1) *has an anti-periodic mild solution.*

### *Proof*

Let $D=\{u\in P_{TA}(R,X_{0})\cap C_{h}(X_{0}): \Vert u\Vert \le r\}$, and $D(t):=\{\Gamma u:u\in D\}$. We define the operator $\Gamma$ by

$$\begin{aligned} \Gamma u(t) = \int _{-\infty }^{t}T_{-1}(t-s)f(s,u(s))ds. \end{aligned}$$

We divide the proof in several steps.

Step 1. For $u\in D$, we have that

$$\begin{aligned} \left\Vert \Gamma u(t) \right\Vert&= \left\Vert \int _{-\infty }^{t}T_{-1}(t-s)f(s,u(s))ds\right\Vert \nonumber \\ &\le M\int _{-\infty }^{t}e^{\omega (t-s)}\left\Vert f(s,u(s))\right\Vert ds\nonumber \\ &\le M\int _{-\infty }^{t}e^{\omega (t-s)}W(\left\Vert u(s)\right\Vert ) ds\nonumber \\ &\le M\int _{-\infty }^{t}e^{\omega (t-s)}W(\left\Vert u\right\Vert _{h}h(s)) ds\nonumber \\ &\le M\beta (\left\Vert u\right\Vert _{h})\nonumber \\ &\le r.\nonumber \end{aligned}$$

So $\left\Vert \Gamma u\right\Vert \le r$. It follows from condition $(A_{2})$ that $\Gamma :C_{h}(X_{0})\rightarrow C_{h}(X_{0})$.

Step 2. The map $\Gamma$ is continuous. In fact, for $\epsilon >0$, we take $\delta$ involved in condition $(A_{3})$. If $u,v\in C_{h}(X_{0})$ and $\Vert u-v\Vert _{h}\le \delta$, then

$$\begin{aligned} \left\Vert \Gamma u(t)-\Gamma v(t)\right\Vert _{h}&= {} \frac{ \left\Vert \int _{-\infty }^{t}T_{-1}(t-s)[f(s,u(s))-f(s,v(s))]ds\right\Vert }{h(t)} \nonumber \\ &\le \frac{M\int _{-\infty }^{t}e^{\omega (t- s)}\Vert f(s,u(s))-f(s,v(s))\Vert ds}{h(t)}\nonumber \\ &\le M\epsilon ,\nonumber \end{aligned}$$

which shows the assertion.

Step 3. We will show that $\Gamma$ is a compact operator.

We will prove that $D(t):=\{\Gamma u:u\in D\}$ is a relatively compact subset of $X_{0}$ for each $t\in R$.

For each $t\in R$, $0<\varepsilon <1$, define

$$\begin{aligned} \Gamma _{\varepsilon } u &= \int _{-\infty }^{t-\varepsilon }T_{-1}(t-s)f(s,u(s))ds \nonumber \\ &= T_{0}(\varepsilon ) \int _{-\infty }^{t-\varepsilon }T_{-1}(t-\varepsilon -s)f(s,u(s))ds \nonumber \\ &= T_{0}(\varepsilon ) \Gamma u(t-\varepsilon ).\nonumber \end{aligned}$$

Since $\{\Gamma u(t-\varepsilon )\}$ is bounded and $T_{0}(\varepsilon )$ is compact, $\{\Gamma _{\varepsilon } u, u\in D\}$ is a relatively compact subset of $X_{0}$, then

$$\begin{aligned} \Vert \Gamma u-\Gamma _{\varepsilon } u\Vert &= \left\Vert \int _{t-\varepsilon }^{t}T_{-1}(t-s)f(s,u(s))ds\right\Vert \nonumber \\ &\le {} \int _{t-\varepsilon }^{t}M e^{\omega (t- s)}\Vert f(s,u(s))\Vert ds \nonumber \\ &\le M\int _{t-\varepsilon }^{t}e^{\omega (t-s)}W(\Vert u(s)\Vert ) ds\nonumber \\& \le {} M\int _{t-\varepsilon }^{t}e^{\omega (t-s)}W(\Vert u(s)\Vert _{h}h(s)) ds.\nonumber \\ &\le M W(\Vert u(s)\Vert _{h}h(s))\varepsilon .\nonumber \end{aligned}$$

So $$\Vert \Gamma u-\Gamma _{\varepsilon } u\Vert _{h}\rightarrow 0$$, as $$\varepsilon \rightarrow 0$$.

Thus, $D(t):=\{\Gamma u:u\in D\}$ is a relatively compact subset of $X_{0}$ for each $t\in R$.

Next,we will show that the set *D* is equicontinuous.

In fact, proceeding as above, for $t_{1}<t_{2}$, $t_{1},t_{2}\in R$, we can decompose

$$\begin{aligned} \Gamma u(t_{2})-\Gamma u(t_{1})&= {} \int _{-\infty }^{t_{2}}T_{-1}(t_{2}-s)f(s,u(s))ds- \int _{-\infty }^{t_{1}}T_{-1}(t_{1}-s)f(s,u(s))ds\nonumber \\&= \int _{-\infty }^{t_{1}-\gamma }(T_{-1}(t_{2}-s)-T_{-1}(t_{1}-s))f(s,u(s))ds\nonumber \\ &\quad + \int _{t_{1}-\gamma }^{t_{1}}(T_{-1}(t_{2}-s)-T_{-1}(t_{1}-s))f(s,u(s))ds\nonumber \\&\quad + \int _{t_{1}}^{t_{2}}T_{-1}(t_{2}-s)f(s,u(s))ds.\nonumber \end{aligned}$$

For each $$\epsilon >0$$,

$$\begin{aligned}& \left\Vert \int _{-\infty }^{t_{1}-\gamma }(T_{-1}(t_{2}-s)-T_{-1}(t_{1}-s))f(s,u(s))ds\right\Vert \nonumber \\&\quad =\left\Vert (T_{0}(t_{2}-t_{1}+\gamma )-T_{0}(\gamma ))\int _{-\infty }^{t_{1}-\gamma }T_{-1}(t_{1}-\gamma -s)f(s,u(s))ds\right\Vert \nonumber \\&\quad \le \left\Vert T_{0}(t_{2}-t_{1}+\gamma )-T_{0}(\gamma )\right\Vert \left\Vert \int _{-\infty }^{t_{1}-\gamma }T_{-1}(t_{1}-\gamma -s)f(s,u(s))ds\right\Vert \nonumber \\&\quad \le \left\Vert T_{0}(t_{2}-t_{1}+\gamma )-T_{0}(\gamma )\right\Vert \int _{-\infty }^{t_{1}-\gamma }Me^{\omega (t_{1}-\gamma -s)}W(\left\Vert u\right\Vert _{h}h(s))ds \nonumber \end{aligned}$$

From step 1, we get $\left\Vert \int _{-\infty }^{t_{1}-\gamma }(T_{-1}(t_{2}-s)-T_{-1}(t_{1}-s))f(s,u(s))ds\right\Vert \rightarrow 0$ as $t_{2}-t_{1}\rightarrow 0$. Moreover, similarly estimates as proof of $\left\Vert \Gamma u-\Gamma _{\varepsilon } u\right\Vert$, when $t_{2}-t_{1}\rightarrow 0$, we can prove that

$$\begin{aligned} \left\Vert \int _{t_{1}-\gamma }^{t_{1}}(T_{-1}(t_{2}-s)-T_{-1}(t_{1}-s))f(s,u(s))ds\right\Vert \rightarrow 0 \end{aligned}$$

and

$$\begin{aligned} \left\Vert \int _{t_{1}}^{t_{2}}T_{-1}(t_{2}-s)f(s,u(s))ds\right\Vert \rightarrow 0. \end{aligned}$$

Combining these estimates, we get

$$\begin{aligned} \left\Vert \Gamma u(t_{2})-\Gamma u(t_{1})\right\Vert \rightarrow 0 \end{aligned}$$

as $t_{2}-t_{1}\rightarrow 0$ and independent of $u\in D$.

Finally, applying condition $(A_{2})$, we can show that

$$\begin{aligned} \frac{\left\Vert \Gamma u\right\Vert }{h(t)}\le \frac{M}{h(t)}\int _{-\infty }^{t}e^{\omega (t-s)}W(rh(s))ds\rightarrow 0,\quad |t|\rightarrow \infty , \end{aligned}$$

and this convergence is independent of $u\in D$.

Hence *D* satisfies conditions (i)–(iii) of Lemma 4, so *D* is a relatively compact set in $C_{h}(X_{0})$. It follows from the proof of steps 1–3 that $\Gamma$ is a compact operator.

Step4. Applying with Theorem 1, we obtain that $\Gamma (P_{TA}(R,X_{0}))\subseteq P_{TA}(R,X_{0})$. Consequently, combining with step 1 and step 2 we infer that $\Gamma (P_{TA}(R,X_{0})\cap C_{h}(X_{0}))\subseteq P_{TA}(R,X_{0})\cap C_{h}(X_{0})$, and also

$$\begin{aligned} \Gamma (\overline{P_{TA}(R,X_{0})\cap C_{h}(X_{0})}^{h})\subseteq \overline{\Gamma (P_{TA}(R,X_{0})\cap C_{h}(X_{0}))}^{h}\subseteq \overline{P_{TA}(R,X_{0})\cap C_{h}(X_{0})}^{h}. \end{aligned}$$

where $\overline{D}^{h}$ denotes the closure of *D* in $C_{h}(X_{0})$. Applying the Lemma 9 (Schauder’s Fixed Point Theorem), we deduce that $\Gamma$ has a fixed point $u\in \overline{P_{TA}(R,X_{0})\cap C_{h}(X_{0})}^{h}$.

Step 5. we prove that $u\in P_{TA}(R,X_{0})$.

Let $(u_{n})_{n}$ be a sequence in $P_{TA}(R,X_{0})\cap C_{h}(X_{0})$ that converges to *u* for the topology in $C_{h}(X_{0})$. It follows from condition $(A_{3})$ that $\Gamma u_{n}\rightarrow \Gamma u$ as $n\rightarrow \infty$, uniformly on *R*. This implies that $u\in P_{TA}(R,X_{0})$, which completes the proof. $\square$

### *Remark 2*

The assumption $(A_{3})$ of Theorem 6 is fulfilled in the following situation:

$$\begin{aligned} \left\Vert f(t,h(t)x)-f(t,h(t)y)\right\Vert \le W(\left\Vert x-y\right\Vert ), \end{aligned}$$

for all $$t\in R$$, $$x,y\in X_{0}$$.

In fact, we use the same notations as in Theorem 6.

$$\begin{aligned} \left\Vert \Gamma u(t)-\Gamma v(t)\right\Vert &= \left\Vert \int _{-\infty }^{t}T_{-1}(t-s)[f(s,u(s))-f(s,v(s))]ds\right\Vert \nonumber \\ &\le M\int _{-\infty }^{t}e^{\omega (t- s)}W\left( \frac{\left\Vert u(s)-v(s)\right\Vert }{h(s)}\right) ds\nonumber \\ &\le \frac{M}{-\omega }W(\left\Vert u-v\right\Vert _{h}).\nonumber \end{aligned}$$

Since *W* is continuous, the above estimate shows that $(A_{3})$ hold, the remains of proof is essentially the same of Theorem 6.

## An example

In this section we give an example to illustrate the above results. Consider the following partial differential equation:

2 $$\begin{aligned} \partial _{t}u(t,x)=\partial _{x}^{2}u(t,x)-\omega u(t,x)+F(t,u(t,x)),\quad t\in R,\ x\in [0,\pi ], \quad \omega <0, \end{aligned}$$

with boundary initial conditions

3 $$\begin{aligned} u(t,0)=u(t,\pi )=0,\quad t\in R. \end{aligned}$$

Let $X=C([0,\pi ],R)$ and the operator *A* be defined on *X* by $Au=u''-\omega u$, with domain

$$\begin{aligned} D(A)=\{u\in X: u''\in X, u(0)=u(\pi )=0\}. \end{aligned}$$

It is well known that *A* is a Hille–Yosida operator of type-$\omega$ with domain nondense (Prato and Sinestrari 1987). The above partial differential equation can be rewritten as an abstract system of the Eq. (1), where $u(t)(s)=u(t,s)$.

Let us consider the nonlinearity $F(t,x)(s) = \beta b(t)\sin (x(s))$ for all $x\in X$ and $s\in [0,\pi ]$, $t\in R$, where *b*(*t*) is a bounded periodic function with period *T*, thus we have

$$\begin{aligned} F(t+T,-x)(s)=\beta b(t+T)\sin (-x(s))=-\beta b(t)\sin (x(s))=-F(t,x)(s) \end{aligned}$$

and

$$\begin{aligned} \left\Vert F(t,x)-F(t,y)\right\Vert =\left\Vert \beta b(t)\sin (x(s))-\beta b(t)\sin (y(s))\right\Vert \le |\beta ||b(t)|\left\Vert x-y\right\Vert . \end{aligned}$$

If $\int _{-\infty }^{t}e^{\omega (t-s)}b(s)ds<\frac{1}{|\beta | M}$, where $\omega <0$. Then the Eq. (2) with boundary initial conditions (3) has a unique anti-periodic mild solutions.

## Conclusions

This paper is concerned with the semilinear differential equation $u'(t) = Au(t) + f (t, u(t))$ with nondense domain. Under some suitable conditions, we establish the existence of anti-periodic (or anti-periodic differentiable) mild solutions to the semilinear differential equation. To the best of our knowledge, it is the first time to deal with this problem. Moreover, the method of this paper can be applied to many other differential equations, such as impulsive differential equations, neutral functional differential equations, fractional differential equations and so on.
